# Supplementary material for: The interactome of intact mitochondria by cross-linking mass spectrometry provides evidence for coexisting respiratory supercomplexes
Source: Mol Cell Proteomics. 2017 Dec 8;17(2):216–32. doi: 10.1074/mcp.RA117.000470 (PMC5795388; doi:10.1074/mcp.RA117.000470)
Supplement: Supplemental Data [file supp_RA117.000470_133922_0_supp_23978_fzffwf.zip › spectra_annotation/mito_DR_spectra_annotation/459-1-19-1-11-1.pdf]

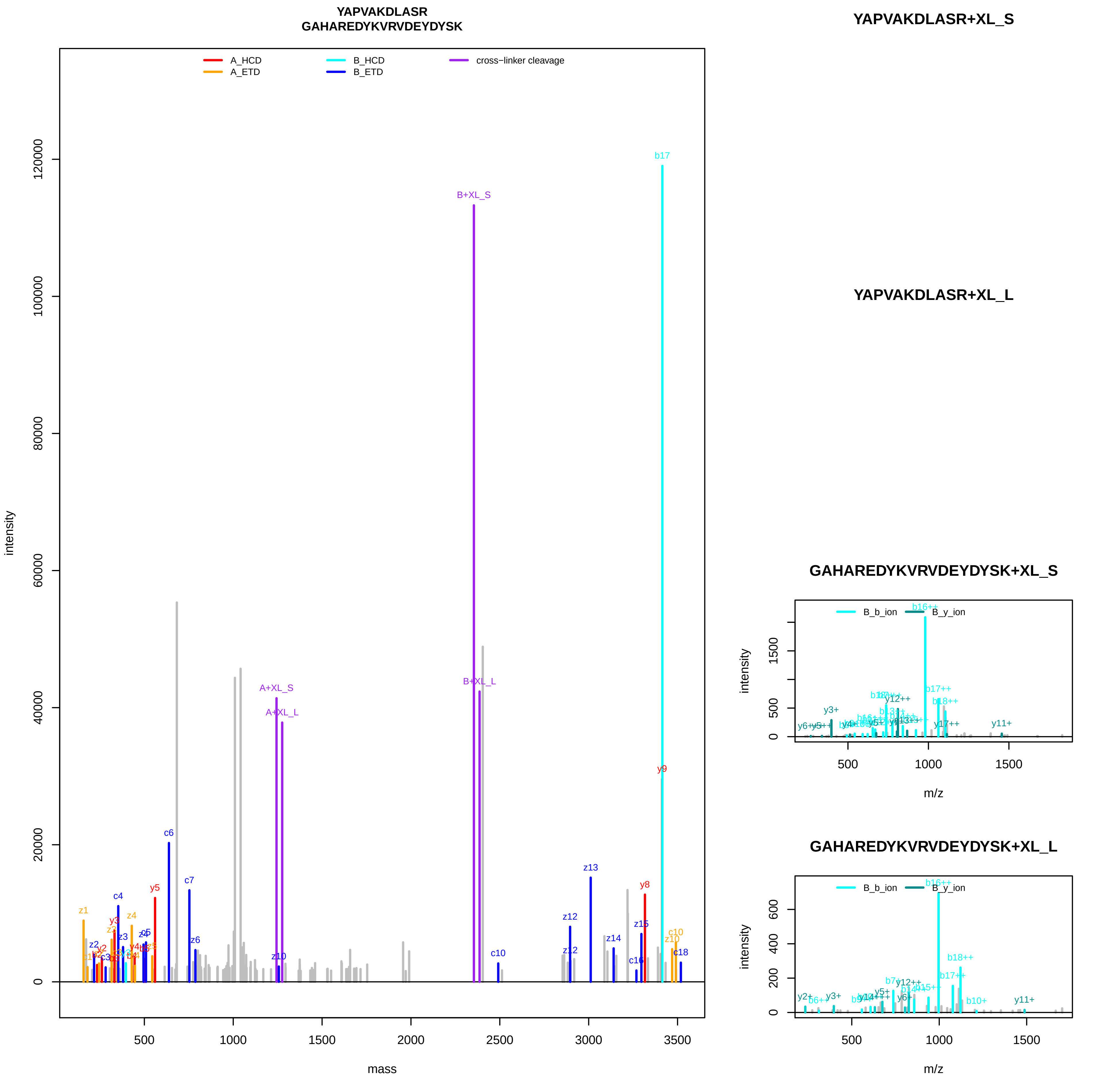
YAPVAKDLASR
GAHAREDYKVRVDEYDYSK
A_HCD
A_ETD
B_HCD
B_ETD
cross−linker cleavage
B+XL_S
B+XL_L
A+XL_S
A+XL_L
b17
y9
y8
y5
c6
c7
c10
c16
c18
c4
z12
z13
z14
z15
z10
c10
intensity
mass
YAPVAKDLASR+XL_S
YAPVAKDLASR+XL_L
GAHAREDYKVRVDEYDYSK+XL_S
B_b_ion
B_y_ion
b16++
b17++
b18++
y12++
y3+
y11+
y17++
intensity
m/z
GAHAREDYKVRVDEYDYSK+XL_L
B_b_ion
B_y_ion
b16++
b18++
b17++
b15++
b7+
y12++
b10+
y11+
y2+
y3+
intensity
m/z
